# Coverage and Drivers to Reaching the Last Child With Vaccination in Urban Settings: A Mixed-Methods Study in Kampala, Uganda

**DOI:** 10.9745/GHSP-D-21-00663

**Published:** 2022-08-30

**Authors:** Carol Kamya, Faith Namugaya, Charles Opio, Paul Katamba, Emily Carnahan, Anne Katahoire, Joanita Nankabirwa, Jaffer Okiring, Peter Waiswa

**Affiliations:** a Infectious Diseases Research Collaboration, Kampala, Uganda.; b PATH, Seattle, WA, USA.; c Makerere University, Kampala, Uganda.; d Uganda and Global Health Division, Karolinska Institutet, Solna, Sweden.

## Abstract

**Background:**

Limited evidence exists regarding the drivers of vaccination coverage and equity in Kampala city, despite frequent measles outbreaks, inequities in vaccination coverage, and the decline in vaccination coverage rates. This study was designed to determine vaccine coverage among children aged 12–36 months and to understand its demand-side drivers.

**Methods:**

We utilized a mixed-methods parallel convergent study design. A household survey was conducted to quantify the drivers of vaccine coverage among households with children aged 12–36 months. We employed a multistage sampling approach to select households, using a primary sampling unit of an enumeration area. We conducted 30 key informant interviews, 7 focus group discussions, and 6 in-depth interviews with representatives from the immunization program, health workers, and parents residing in areas with low vaccine coverage.

**Results:**

Of the 590 enrolled children, 340 (57.6%) were partially vaccinated, 244 (41.4%) were fully vaccinated and had received all the recommended vaccinations, and 6 (1.0%) had never received any vaccine. Of the 244 with all recommended vaccinations, only 65 (26.6%) received their vaccines on time. Access to vaccination services was high (first dose of diphtheria, pertussis, and tetanus [DPT1] coverage of 96%), but utilization decreased over time, as shown by a dropout rate of 17.3% from the first to third dose of DPT. The main driver of complete vaccination was the parents’ appreciation of the benefits of vaccination. Among partially vaccinated children, the barriers to vaccination were inadequate information about vaccination (its benefits and schedule), vaccine stock-outs, long waiting times to receive vaccination services, and hidden vaccination costs.

**Conclusion:**

Vaccination needs to be targeted to all children irrespective of whether they reside in slum areas or nonslum areas, as most are under-vaccinated. Social mobilization and communication efforts should be tailored to the complexities of urban settings characterized by transient and diverse populations with different cultures.

## Abstract (precis)

Most children in Kampala city are not fully vaccinated as the health system is not designed to suit the complex urban setting.

## Key Messages

- Access to vaccination services for children in Kampala is high; however, vaccination uptake and timeliness decrease over time, as indicated by the high percentage of partially vaccinated children (58.6%).
- Parents of partially vaccinated children stated that receiving inadequate information about immunization from health workers was a barrier to vaccination. This highlights the need for tailored health education and social mobilization efforts in Kampala taking into consideration the transient and diverse populations.
- Vaccine stock-outs and long waiting times prevented parents from vaccinating their children. Stronger public-private partnerships in urban areas could help address these barriers.
- A primary health care model that harnesses the strengths of the private sector can help address the barrier of hidden immunization costs that deter people from seeking immunization services.
- Innovative survey methods that capture data from the highly mobile nonresidents in urban settings need to be developed as this population contributes to the pool of children that need to be immunized but also to the risk of disease outbreaks.

## BACKGROUND

Fifty-five percent of the world’s population in 2018 was estimated to live in urban areas.^1^ This percentage is projected to increase to 68% by 2050, with Asia and Africa urbanizing most rapidly.^1^ Urbanization positively affects economic growth, poverty reduction, and human development, but it leads to increased population density in urban slums.^1^ Moreover, substantial disparities in health exist between people residing in slums and those in nonslum environments, exacerbating children’s vulnerability to infections.^2^ Urban slums have lower vaccine coverage and large pockets of unvaccinated children.^3^ Urban settings pose unique barriers to the delivery and utilization of vaccination services owing to the highly transient populations, parents’ inflexible work schedules, a mix of private and public providers, unfavorable vaccination session times, and a culturally diverse population.^3^^,^^4^

The World Health Organization’s (WHO’s) Expanded Program on Immunization (EPI) was designed over 40 years ago to overcome geographic barriers to accessing services for rural populations, and it has successfully helped increase vaccination coverage rates. However, with increasing urbanization, the focus needs to be adapted to urban environments to meet the needs of a growing urban population.^5^ The Global Vaccine Action Plan 2011 aimed for more equitable coverage, but national and global responses did not address emerging issues such as urbanization.^6^^,^^7^ The Immunization Agenda 2030 proposes to increase equitable access and use of new and existing vaccines even with emerging challenges, including urbanization, migration conflict, and climate change.^7^

In Uganda, the population living in urban areas has increased steadily from 18% in 2008 to 24% in 2018.^8^ Uganda’s metropolitan areas have poor vaccination coverage, often resulting in outbreaks of vaccine-preventable diseases. Kampala is Uganda’s capital and its largest city, with an annual population growth rate of 3%.^9^ Sixty percent of the population resides in slum areas.^10^ In addition, Kampala hosts refugees and asylum seekers from South Sudan, the Democratic Republic of Congo, Burundi, Somalia, Rwanda, and other countries.^11^^,^^12^ The ever-increasing urban population is characterized by densely populated substandard houses, social and economic isolation, irregular land ownership, low standards of sanitation, and limited access to basic infrastructure and social services.^5^^,^^13^

Persistent challenges in the coverage and equity of vaccination are present in Kampala. The Uganda Health Management Information System reports a high full vaccination coverage of over 90% for Kampala city.^14^ However, surveys show a decline in full vaccination coverage from 77% in 2010 to 51% in 2016 and 48% in 2017.^14^^–^^17^ In addition, a 2016 vaccination equity assessment and the occurrence of several measles outbreaks indicate a high number of unimmunized or under-immunized children in the city.^18^^,^^19^ Vaccination coverage is influenced by demand-side drivers (how individuals seek, access, and utilize services) and supply-side drivers (how services are communicated and delivered).

A few studies have examined the barriers and facilitators of vaccination coverage and equity in Uganda, specifically in urban settings. A study conducted in 2010 in Kampala showed that vaccination behavior was affected by the negative influence of male partners, the need to have presentable clothing at vaccination visits, inconvenient vaccination clinic schedules, and suspicion about vaccines.^20^ A separate study identified health system barriers to childhood vaccination in Kampala, including poor geographical access to vaccination facilities, long waiting times at health facilities, intermittent availability of vaccines, and “informal” fees for vaccination.^21^ Evidence from other countries shows that key drivers to the vaccination include trust of health care providers, which is linked to open communication; provision of free vaccination services; availability of vaccine logistics; and belief that vaccines are effective.^22^^–^^24^ Meanwhile, barriers include the lack of adequate information about vaccination and potential adverse events, long distances to vaccination points, spousal pressure not to vaccinate, negative health worker attitude, and long wait time.^23^^–^^25^

Considering the declining vaccination coverage in Kampala, the increasing number of outbreaks, and high number of under-vaccinated children, the factors that facilitate or hinder optimal vaccine coverage and equity in Kampala city are unclear. The situation has been exacerbated by the coronavirus disease (COVID-19) pandemic, which has significantly affected the continuity of primary health care services, particularly vaccination.^26^ Disruption of vaccination services has increased the risk of vaccine-preventable diseases and outbreaks. Understanding vaccine coverage and the barriers to reaching the last child is vital, especially in urban settings. We sought to determine the vaccine coverage among children aged 12–36 months living in Kampala and to understand the demand-side drivers of vaccination coverage in Kampala city. The study was conducted before the pandemic and focused on childhood routine vaccination, but the findings can have important implications for vaccination across the lifecycle, including COVID-19 vaccination uptake.

Understanding vaccine coverage and the barriers to reaching the last child is vital, especially in urban settings.

## METHODS

### Study Design

We used a mixed-methods parallel convergent study design.^27^^,^^28^ Complementary quantitative and qualitative data were collected concurrently but analyzed separately. Quantitative data were collected through a household survey. Qualitative data were collected through key informant interviews (KIIs), fact-checking interviews, focus group discussions (FGDs), and in-depth interviews (IDIs). Quantitative and qualitative results were interpreted jointly to triangulate findings. Fact-checking interviews were subsequently conducted to validate the findings.

### Study Setting

This study was conducted in Kampala from June 2019 to May 2020. Kampala is Uganda’s most densely populated city and a major regional trade and transport hub. The city has 5 administrative regions—Central, Kawempe, Makindye, Rubaga, and Nakawa divisions—and its population is approximately 1.6 million people at night and 4.5 million during the day.^17^

Kampala city has 1,448 public and private health facilities. Of these, 301 (21%) offer vaccination services. The majority (284/301) of those health facilities are private. The Uganda National Expanded Program on Immunization (UNEPI) manages vaccination in Uganda with support from partners.^29^ The Kampala City Council Authority Department of Public Health and Environment is mandated to provide high-quality vaccination services in Kampala. According to Uganda’s vaccination schedule, children receive the following vaccines: (1) bacille Calmette-Guérin (BCG) and oral polio vaccine at birth (polio0); (2) first dose of diphtheria, pertussis, and tetanus vaccine (DPT1), first dose of pneumococcal conjugate vaccine (PCV1), rotavirus, and oral polio at 6 weeks (polio1); (3) second doses of DPT, PCV and (DPT2, PCV2), and polio (polio2) at 10 weeks; (4) third doses of DPT (DPT3), PCV (PCV3), and inactivated polio vaccine (IPV) at 14 weeks; and (5) measles at 9 months.^30^

### Quantitative Component

#### Household Survey

We conducted a household survey among parents of children aged 12–36 months in all divisions of the city between September and November 2019. For children aged 12–23 months, coverage was measured for 1 year before the survey, and for children aged 24–36 months, coverage was measured for 2 years before the survey.

The primary outcome was the proportion of children with DPT3 vaccination. Secondary outcomes were the proportions of children with BCG, measles, DPT1, PCV, and IPV vaccination. The predictor variables of interest included child, parent, and household characteristics.

#### Sampling Determination and Procedure

To estimate the sample size, we used a difference between 2 independent proportions: (1) children with complete DPT3 vaccination whose family could afford transport fare to seek vaccination services, and (2) those whose family could not afford transport fare to seek vaccination services.^21^^,^^31^ We set the significance level at 5%, power at 80%, a design effect of 2, and a nonresponse rate of 10%. We computed the sample size for 3 key potential drivers, including (1) physical access, as measured by high transport fare to the facility while seeking vaccination services; (2) the need to pay for vaccination services; and (3) poor geographical access to vaccination facilities.^21^^,^^31^ The highest sample size was 553, which was rounded to 600 households. This sample size was based on the proportion of children with complete DPT3 vaccination whose family could afford transport fares to seek vaccination services.

We employed multistage sampling to select the households. Kampala city is divided into 5 divisions and 97 parishes. Each parish includes multiple enumeration areas (EAs). The primary sampling unit was the census EA.^9^ Kampala was divided into 3 sampling strata (lower, middle, and upper) based on income poverty and the number of measles cases: (1) lower-income group: parishes with an income poverty of more than 5.0% and a minimum of 10 measles cases reported in 2017; (2) middle-income group: parishes with an income poverty of 2.5%–5.0% and less than 10 measles cases reported in 2017; and (3) upper-income group: parishes with an income poverty of less than 2.5% and less than 10 measles cases reported in 2017.^9^^,^^32^ The sample then drew from both slum and non-slum areas that were already stratified.

**FIGURE 1:**
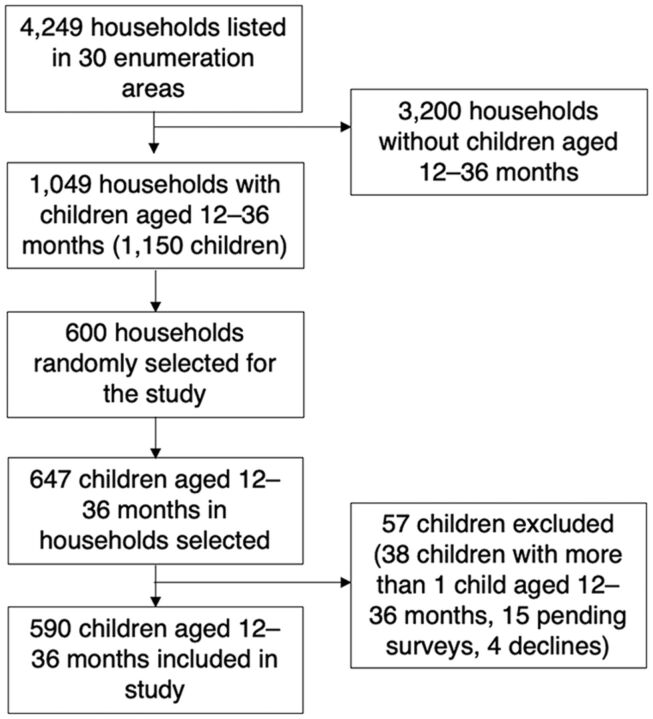
Study Profile Assessing Vaccine Coverage Among Children Aged 12–36 Months, Kampala, Uganda

Based on the Kampala Population and Housing Census conducted in August 2014 by the Uganda Bureau of Statistics, there were 3,297 EAs.^9^ We estimated the optimal number of clusters as the square root of the total number of clusters divided by 2 and determined that 30 EAs were required for the survey. We randomly selected 10 EAs from each of the 3 strata. We listed all the households in each EA to determine the total number of eligible households and to generate a household recruitment list. Using this list, we obtained the number of eligible households for each EA proportionate to the household population and then generated a random list for each EA. Households were then approached in the order of the random list, and a household was enrolled if it had at least 1 child aged 12–36 months. The study profile is summarized in Figure 1. Slums were defined as predominantly residential areas characterized by high population densities, deteriorated buildings, littered streets, unsanitary and hazardous conditions, and economic distress, as defined by the Uganda Bureau of Statistics.^33^

#### Data Collection

We adapted the household survey questionnaire from the Uganda Demographic Health Surveys (UDHS),^17^ WHO, UNICEF urban tool kit, and prior cross-sectional community surveys.^15^ The questionnaire was preprogrammed onto handheld tablet computers, including range and internal checks. The household survey was administered to parents with children aged 12–36 months who had provided informed consent to participate in the study. A household was excluded if it was vacant on 5 visits or if the household head did not provide consent. Vaccination data were collected from vaccination cards.

#### Data Analysis

Data were analyzed using Stata version 14.1 (Stata Corp) and R 3.4.1. We generated descriptive statistics (frequencies, percentages, means) to characterize the study population, and analyses were conducted to describe and illustrate the characteristics.

Principal component analysis was used to generate a wealth index based on ownership of everyday household items. Households were ranked by wealth scores and grouped into 4 tertiles to obtain a categorical measure of socioeconomic position. We conducted weighted (using svy: command) mixed-effects bivariate and multivariable logistics regression analyses to identify the determinants of full vaccination coverage.

Complete vaccination was defined as a child having received all 13 doses on Uganda’s vaccination schedule: BCG, DPT1, DPT2, DPT3, PCV1, PCV2, PCV3, IPV, measles, Polio0, Polio1, Polio2, and Polio3. Partial vaccination was defined as a child having received some vaccines but not all vaccinations on the schedule. Unvaccinated was defined as a child not having received any antigen dose. Timely vaccination was defined as completion of the recommended vaccination schedule within WHO’s recommended time ranges: (1) BCG from birth to 8 weeks; (2) polio0 from birth to 4 weeks; (3) DPT1, PCV1, and polio1 from 4 weeks to 2 months; (4) DPT2, PCV2, and polio2 from 8 weeks to 4 months; (5) DPT3, PCV3, and polio3 from 12 weeks to 6 months; and (6) measles vaccine from 38 weeks to 12 months.^34^ Measures of association were expressed as odds ratios (ORs).

### Qualitative Component

#### Data Collection

Document reviews were conducted to understand the main challenges of vaccine service delivery in Kampala city and how UNEPI is adapting to these challenges. Documents reviewed included Gavi, the Vaccine Alliance guidance documents and reports; Ministry of Health/UNEPI reports; and Uganda national plans and strategies, policies, guidelines, presentations, and published articles. Findings from the document review informed the collection of data through KIIs.

Thirty KIIs were conducted at global, national, and community levels to gain insights into stakeholder perspectives on delivering vaccination services in Kampala. Key informants were purposively selected based on their knowledge and experience in urban health. Data were collected using a semistructured KII guide about perceived drivers of low vaccination coverage in Kampala city. Key informants were drawn from UNEPI; village health team (VHT) members; Kampala City Council Authority; UNEPI focal persons at the district level; and representatives from development partners, including United Nations agencies, bilateral agencies, and international nongovernmental organizations.

To understand community awareness and perceptions regarding vaccination, especially among parents of unvaccinated and partially vaccinated children, FGDs were conducted with parents in areas with low vaccine coverage, including Indian communities, slums, refugee communities (Somali, south Sudanese settlements), and Indian residences. FGD participants were identified through secondary data analysis of vaccination coverage data and KIIs conducted with VHT members and UNEPI focal persons. Using a semistructured discussion guide, we conducted 7 FGDs among parents with children aged 12–36 months. Questions were posed in an open-ended manner, followed by specific prompts. The FGDs explored the reasons why parents were not vaccinating their children.

We conducted 6 IDIs with parents whose children were partially immunized or unimmunized to gain a more in-depth understanding of the issues that surround children not being vaccinated. IDI respondents were identified through interviews with VHT members. Data were collected using a discussion guide focusing on the reasons why children were partially vaccinated or unvaccinated. IDIs lasted about 1 hour. Fact-checking interviews were conducted with UNEPI, Kampala City Council Authority, UNEPI focal persons, and VHTs to validate findings.

#### Qualitative Data Analysis

All the IDIs, KIIs, and FGDs were audio-recorded and transcribed. If they were conducted in the local language, they were also translated into English. The transcripts were imported and managed in NVIVO software (QSR International, qualitative data analysis software). We employed a thematic content analysis for the qualitative data, with 2 research team members doing the analysis. They began by reading through the transcripts and assigning preliminary codes to the data to describe the content. Both team members read all transcripts. The analysis was primarily deductive, using a coding framework informed by the Social Ecological Model.^35^ The 2 research team members searched for patterns or themes in the codes across the different interviews based on the model. All research team members then agreed upon the themes. Data were categorized as representing either facilitators or barriers to vaccination service delivery. Coding was flexible, with new codes being added and existing codes modified inductively based on the data to allow for unique themes to emerge. We also employed root cause analysis to identify causal factors underpinning a chain of events.^36^ This approach was used to uncover the reasons underlying challenges and successes. We conducted root cause analyses using secondary and primary data sources to test and refine assumptions about causal pathways based on consensus by the study team.

### Ethical Considerations

The study was approved by the Makerere University School of Biomedical Sciences Research and Ethics Committee and the Uganda National Council for Science and Technology ethics committee. Written informed consent was obtained from all study participants before participation in the study.

## RESULTS

### Sociodemographic Characteristics

Between September 2019 and November 2019, 4,249 households were listed and visited, and, of those, 1,049 (25%) had children aged 12–36 months. We randomly selected 600 households for the study, which included 647 children. A total of 590 (91%) of these children were included in the study (Figure 1).

Table 1 presents details of the characteristics of the study participants. The median age of the children at enrollment was 2.1 years (standard deviation of 0.7). Over half of the children were male (n=306, 51.9%). Most of the children (n=561, 95.1%) were delivered at a health facility with vaccination services and were Christian (n=433, 73.4%). Many of the children were living in households headed by males (n=439, 74.4%) who were aged younger than 30 years (n=334, 56.5%). Many household heads had attained at least secondary education (n=374, 63%).

**TABLE 1.** Characteristics of Parents and Their Children Aged 12–36 Months That Participated in the Study in Kampala

	**Total, No. (%) (N=590)**	**Unvaccinated, No. (%) (N=6)**	**Partially Vaccinated, No. (%) (N=340)**	**Completely Vaccinated, No. (%) (N=244)**
Sex of the child				
Male	305 (51.7)	3 (50.0)	175 (51.5)	127 (52.1)
Female	285 (48.3)	3 (50.0)	165 (48.5)	117 (47.9)
Religion				
Christian	433 (73.4)	5 (83.3)	252 (74.1)	176 (72.1)
Muslim	136 (23.1)	1 (16.7)	73 (21.5)	62 (25.4)
Other	21 (3.5)	0 (0.0)	15 (4.4)	6 (2.5)
Place of delivery				
Public facility	493 (83.6)	2 (33.3)	280 (82.4)	211 (86.5)
Private facility	68 (11.5)	2 (33.3)	44 (12.9)	22 (9.0)
Home/traditional birth attendant	29 (4.9)	2 (33.3)	16 (4.7)	11 (4.5)
Relationship to child				
Mother	99 (16.8)	1 (16.7)	53 (15.6)	45 (18.4)
Father	256 (43.4)	1 (16.7)	153 (45.0)	102 (41.8)
Other	235 (39.8)	4 (66.6)	134 (39.4)	97 (39.8)
Sex of parents				
Male	39 (6.6)	0 (0.0)	19 (5.6)	20 (8.2)
Female	551 (93.4)	6 (100.0)	321 (94.4)	224 (91.8)
Parents’ education level				
Primary or lower	216 (36.6)	3 (50.0)	138 (40.6)	75 (30.7)
Secondary	208 (35.3)	2 (33.3)	112 (32.9)	94 (38.6)
Tertiary	166 (28.1)	1 (16.7)	90 (26.5)	75 (30.7)
Parent’s age, years				
Younger than 30	334 (56.6)	4 (66.6)	197 (57.9)	133 (54.5)
30–40	203 (34.4)	1 (16.7)	111 (32.7)	91 (37.3)
Older than 40	53 (10.0)	1 (16.7)	32 (9.4)	20 (8.2)
Wealth index				
Poor	298 (50.5)	3 (50.0)	189 (55.6)	106 (43.4)
Less poor	292 (49.5)	3 (50.0)	151 (44.4)	138 (56.6)
Division				
Central	36 (6.1)	0 (0.0)	22 (6.5)	14 (5.8)
Kawempe	93 (15.8)	1 (16.7)	51 (15.0)	41 (16.8)
Rubaga	186 (31.5)	4 (66.6)	109 (32.1)	73 (29.9)
Makindye	93 (15.8)	0 (0.0)	32 (9.4)	61 (25.0)
Nakawa	182 (30.8)	1 (16.7)	126 (37.0)	55 (22.5)

### Vaccination Coverage

**FIGURE 2:**
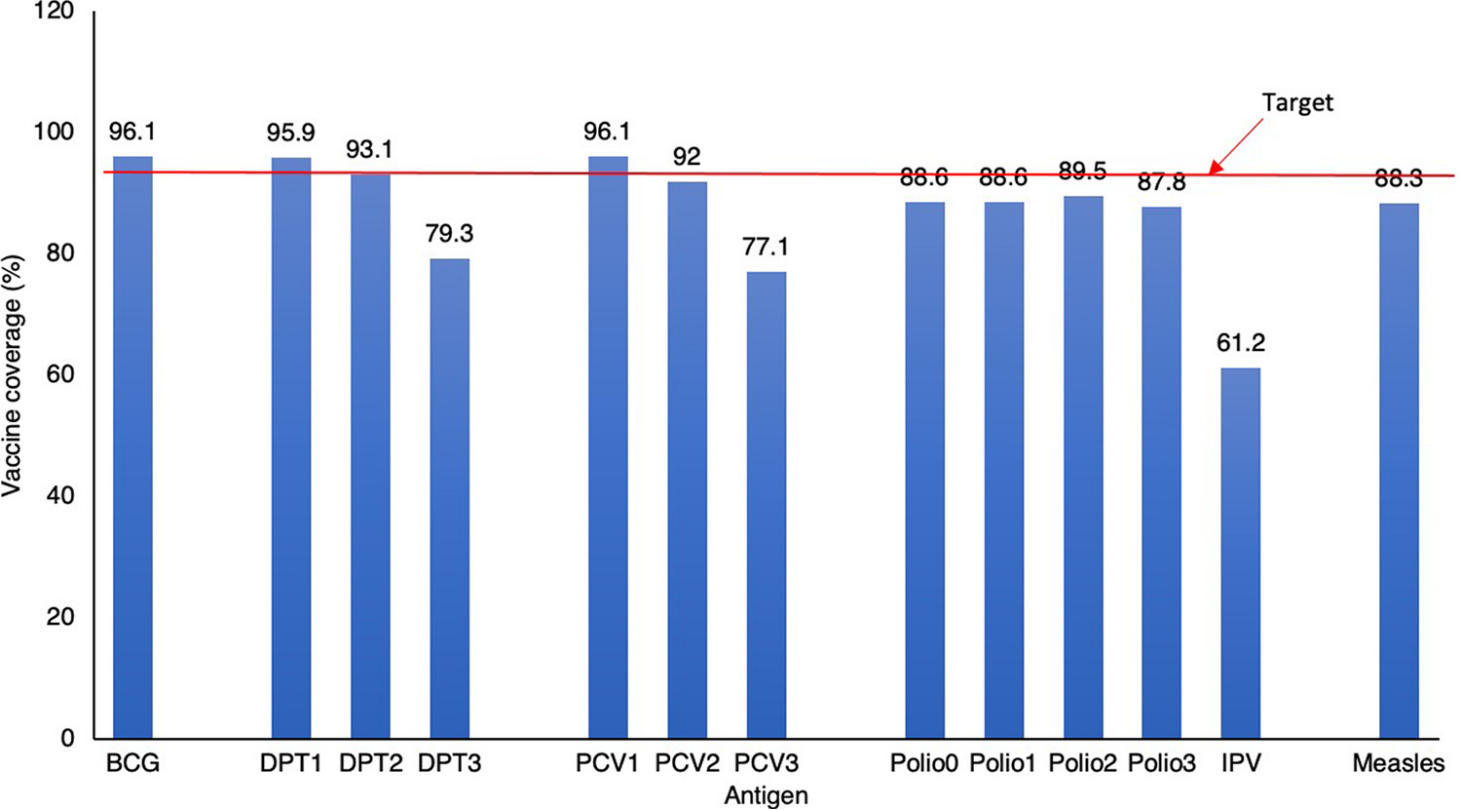
Vaccine Coverage Among Children Aged 12–36 Months in Kampala, Uganda Abbreviations: BCG, bacille Calmette-Guérin; DPT1, first dose of diphtheria, pertussis, and tetanus vaccine; DPT2, second dose of DPT; DPT3, third dose of DPT; IPV, inactivated polio vaccine; PCV1, first dose of pneumococcal conjugate vaccine; PCV2, second dose of PCV; PCV 3, third dose of PCV; polio0, oral polio vaccine at birth; polio1, first dose of polio vaccine; polio2, second dose of polio vaccine; polio3, third dose of polio vaccine.

Of the enrolled children, 340 (57.6%) were partially vaccinated, 244 (41.3%) were fully vaccinated, and 6 (1.0%) had never received any vaccine. Of the 244 that received all recommended vaccinations, only 65 (26.6%) received them on time. The vaccines with the highest coverage were BCG and DPT1 (96.1% and 95.9%, respectively), followed by PVC1 at 95% (Figure 2). IPV had the lowest coverage at 61.2%. A 17.3% dropout rate was observed in the number of children that received DPT1 to those that received DPT3 (96% versus 79.3%, *P*<.001).

We compared the characteristics of fully and unvaccinated or partially vaccinated children in the sample (Table 2). Children with complete vaccination were less likely to be in low-income families than those who were partially vaccinated (56.6% among fully vaccinated children versus 44.5% among partially vaccinated children, *P*=.003). Partially vaccinated children were found in slums (41.8%) and in nonslum areas (40.9%). The highest proportions of fully vaccinated children resided in Rubaga and Makindye divisions (29.9% and 25.1%, respectively), and the highest numbers of partially vaccinated children resided in the Nakawa division (36.7%) and Rubaga division (32.1%). Other characteristics were similar between the fully vaccinated and the unvaccinated or partially vaccinated children. We found no significant differences in vaccination coverage between residences around slum areas (OR: 1.05; 95% confidence interval [CI], 0.67, 1.66).

**TABLE 2.** Characteristics of Completely Vaccinated Children Compared to Unvaccinated or Partially Vaccinated Children in Kampala

	Unvaccinated or Partially Vaccinated, No. (%) (n=346)	Completely Vaccinated, No. (%) (n=244)	Bivariate		Multivariate	
			Odds Ratio (95% CI)	*P* Value	Odds Ratio (95% CI)	*P* Value
Wealth index						
Poor	192 (55.5)	106 (43.4)	Reference		Reference	-
Less poor	154 (44.5)	138 (56.6)	1.71 (1.23, 2.39)	0.003	1.98 (1.24, 2.43)	0.001***
Residence						
Non-slum	169 (48.8)	117 (48.0)	Reference		Reference	-
Slum	177 (51.2)	127 (52.0)	0.99 (0.61, 1.64)	0.992	1.53 (0.88, 2.69)	0.128
Division						
Central	22 (6.4)	14 (5.7)	Reference		Reference	
Kawempe	52 (15.0)	41 (16.8)	1.23 (0.93, 1.63)	0.143	1.99 (0.52, 1.90)	0.976
Rubaga	113 (32.7)	73 (29.9)	0.97 (0.66, 1.43)	0.886	0.89 (0.38, 2.10)	0.781
Makindye	32 (9.2)	61 (25.0)	2.90 (1.74, 4.83)	<0.001	1.96 (1.00, 3.86)	0.050*
Nakawa	127 (36.7)	55 (22.5)	0.66 (0.33, 1.32)	0.231	0.48 (0.14, 1.62)	0.226
Sex of the household head						
Male	268 (77.5)	171 (70.1)	Reference		Reference	
Female	78 (22.5)	73 (29.9)	1.50 (0.92, 2.43)	0.097	1.53 (0.93, 2.53)	0.094
Sex of the child						
Male	178 (51.4)	127 (52.0)	Reference		Reference	
Female	168 (48.6)	117 (48.0)	1.13 (0.74, 1.73)	0.545	1.01 (0.64, 1.59)	0.976
Primary parent’s age, years						
Younger than 20	25 (7.2)	9 (3.7)	Reference		Reference	
21–30	202 (58.4)	138 (56.6)	2.01 (0.77, 5.28)	0.149	0.54 (0.08, 3.48)	0.499
31–40	84 (24.3)	74 (30.3)	2.63 (0.81, 8.47)	0.102	0.57 (0.08, 4.29)	0.571
Older than 40	35 (10.1)	23 (9.4)	1.98 (0.67, 5.88)	0.206	0.56 (0.08, 3.69)	0.530
Number of antenatal care visits (n=573)						
Less than 4 times	86 (25.6)	63 (26.6)	Reference		Reference	
4 times and more	250 (74.4)	174 (73.4)	1.28 (0.86, 1.90)	0.209	1.36 (0.77, 2.43)	0.280
Religion						
Catholic	117 (33.8)	81 (33.2)	Reference		Reference	
Anglican	96 (27.8)	61 (25.0)	0.93 (0.66, 1.32)	0.686	0.81 (0.51, 1.28)	0.354
Muslim	74 (21.4)	62 (25.4)	1.05 (0.71, 1.57)	0.799	0.84 (0.47, 1.52)	0.561
Pentecostal	44 (12.7)	34 (13.9)	1.04 (0.59, 1.81)	0.894	1.02 (0.54, 1.94)	0.945
Others	15 (4.3)	6 (2.5)	0.70 (0.43, 1.13)	0.137	0.17 (0.01, 2.12)	0.162
Education level of household head						
At most primary	86 (24.9)	47 (19.3)	Reference		Reference	
At least secondary	260 (75.1)	197 (80.7)	1.59 (1.01, 2.51)	0.045	1.29 (0.72, 2.31)	0.369
Parity (n=396)						
4 or less	203 (89.4)	157 (92.9)	Reference		Reference	
Greater than 4	24 (10.6)	12 (7.1)	0.58 (0.21, 1.62)	0.289	0.52 (0.19, 1.40)	0.184
Place of delivery						
Public facility	24 (6.9)	211 (86.5)	Reference		Reference	
Private facility	282 (81.5)	22 (9.0)	0.72 (0.42, 1.25)	0.235	0.74 (0.45, 1.24)	0.242
Home/traditional birth attendant	38 (11.6)	11 (4.5)	0.87 (0.21, 3.55)	0.840	1.52 (0.31, 7.37)	0.591
Distance to the health facility						
Less than 20 meters	153 (44.2)	114 (46.7)	Reference		Reference	
20–50 meters	87 (25.1)	56 (23.0)	0.81 (0.50, 1.31)	0.374	0.77 (0.44, 1.35)	0.349
More than 50 meters	7 (2.0)	14 (5.7)	3.10 (1.28, 7.58)	0.015	2.11 (0.41, 10.90)	0.361
Outside Kampala	99 (28.6)	60 (24.6)	0.77 (0.59, 0.99)	0.049	0.95 (0.64, 1.42)	0.791
Transportation costs						
None	194 (56.1)	134 (54.9)	Reference		Reference	
< 5000	128 (37.0)	99 (40.6)	1.27 (0.95, 1.71)	0.104	1.72 (0.89, 3.33)	0.102
> 5000	24 (6.9)	11 (4.5)	0.91 (0.42, 1.97)	0.811	0.99 (0.28, 3.49)	0.990

Abbreviation: CI, confidence interval.

### Facilitators of Vaccination

Of the 244 children that were completely vaccinated, almost all parents (99%) reported that understanding the role of vaccination in protecting their children from disease motivated them to ensure that their children received all the vaccines. Some parents (20.5%) reported that the fear of contracting diseases portrayed on television motivated them to ensure that their children were completely vaccinated. A few (0.8%) believed that vaccination is a child’s right. In all FGDs, community members emphasized that the main reason for taking their children for vaccination was for them to acquire better immunity. Based on personal experiences, they noted that certain diseases such as polio that were common in the past are now rarely seen because of vaccination. In an FGD with women, a mother of an immunized child described how her child who had contracted measles was well enough to go to school, unlike another child that was not immunized.

*I live with my sister’s child, but the child has suffered from measles now and then. But my child, whom I took for vaccination, got measles, but he was strong and even went to school. I was just sympathetic to let him stay home because I didn’t want him to spread the disease to other children at school.* —FGD, women residing in an area with low vaccine coverage

### Barriers to Vaccination

Parents with unvaccinated or partially vaccinated children were asked to identify barriers to receiving vaccination services (Table 3). Nearly all parents (n=344, 99%) cited a lack of information on when their children should receive the subsequent vaccine as the reason they had missed some or all of the scheduled vaccinations.

**TABLE 3.** Reasons Given by Parents for Partially Vaccinating Their Children Aged 12–36 Months in Kampala

**Reason**	**Frequency (%) (n=346)**
Inadequate information on immunization	344 (99.4)
Vaccine stock-outs	71 (20.5)
Child not living with mother	60 (17.3)
Long waiting time	55 (15.9)
Hidden costs	18 (5.2)
Discrimination of minority groups	2 (0.6)

Nearly all parents cited a lack of information on when children should receive subsequent vaccine as the reason they had missed some or all of the scheduled vaccinations.

In an FGD with men living in the slums, respondents mentioned that health workers emphasize the need for follow-up visits but provide no explanations about the vaccines received and their benefits.

*They (health workers) do not tell us why they are immunizing. They just tell us the government has decided that we take our children for immunization, but they do not first sensitize us about its benefits.* —FGD with men residing in slums

Other common reasons cited for missing vaccination were vaccine stock-outs at the health facilities (71, 20.5%), long waiting times at the health facilities (55, 15.9%), and hidden costs associated with vaccination (18, 5.2%).

District officials and UNEPI focal persons cited frequent stock-outs of vaccines at national, district, and health facility levels at private and public facilities as another barrier to vaccination. Key informants mentioned stock-outs of PCV, BCG, and polio antigens.

*The truth is there are stock-outs of vaccines commonly for measles, PCV, and even BCG and OPV. The stock-outs are at 2 levels: health facilities and the Division Vaccine Stores. When there are stock-outs, there is a lot of rationing that takes place.* —KII, district level

*You go to the district vaccine store, and they tell you that the national medical stores are having stock issues—and this is what they have not supplied. When we request for the vaccines from the national medical stores, we often request for enough to use in a month, but we often don’t get what we requested for.* —KII, UNEPI focal person

Parents also reported long waiting times at health facilities deterring them from vaccinating their children, as illustrated by the following extracts from an IDI with a community member and an FGD with women residing in an area with low vaccine coverage.

*The health workers start late, sometimes you go a health facility by 8:00 AM, but the health worker comes in at 10:00 AM or sometimes 11:00 AM*. —IDI, community member

*Parents find it hard because the provider will not immunize your child until the number of children around is enough to use up all the doses in the bottle. So, you will get to the facility and sit there waiting for more children to come, yet we also have other things to do at home.* —FGD, women residing in an area with low vaccine coverage

Furthermore, another common barrier to vaccination among parents was the hidden costs associated with vaccination. These costs included payment for vaccination cards, vaccination services, and special requirements at vaccination points, such as diapers for the children, as illustrated by the following extracts from an IDI with a VHT member and an FGD with women.

*Even selling of the cards as you see. They are selling them at 5,000 shillings (US$1.30). Just because a mother doesn’t have 5,000 shillings (US$1.30) to buy an vaccination card, she is chased away.* —IDI, village health team

*Yes, and yet some people do not even have money for transport to go to the health center for immunization. And, you need to have diapers for the child when you take them to the facility because they may pass out urine.* —FGD, women

Another common barrier to immunization among parents was the hidden costs associated with vaccination.

Parents also cited several other factors that influenced uptake of vaccination services but were not captured in the survey, such as refusal by a spouse, death of a child perceived to be linked to vaccination, and media reports of fake vaccines. These factors resulted in fear and mistrust of vaccines, as shown by the following extracts from interviews and FGDs.

*I also agree with what that lady has said. I also do not immunize my children. My husband refused to immunize our children. I have eight children, and they are all well and healthy, and yet I have never immunized any of them.* —FGD, women

*At that time there was a rampant epidemic of measles in the 1990s. However, many of the children who were vaccinated died. For instance, I lost my child a few months after she was vaccinated, in my case. Most people fear that the same scenario may happen again in this area. Over eight children that I knew died in this area.* —IDI, resident of a slum area

*Yes, in all the newspapers, there have been stories that the doctors are immunizing children with fake vaccines. In the international news … where they said that some European organizations are trying out some vaccines to see if they work. But they are not trying it out on their people, but they have sent it to Africa and mentioned some countries.* —KII, leader residing in an area with low vaccine coverage

## DISCUSSION

Our results show low complete vaccination coverage for children aged 12–36 months in Kampala (41.3%), with more than half (58.6%) partially vaccinated or unvaccinated. In addition, very few children (27%) got vaccinations on time. The main barrier to vaccination in Kampala was inadequate information on the benefits of vaccination. Other obstacles to vaccination included vaccine stock-outs, long waiting times at health facilities, and hidden costs associated with accessing vaccination services. A survey conducted in 2010 estimated that 77.2% had completed the vaccine schedule in Kampala.^15^ In comparison, more recent estimates from the 2016 UDHS reported a 51% vaccination coverage for Kampala.^17^ That report and the current findings indicate a high number of partially vaccinated children. Consistent with our findings, studies conducted in Kenya and India have also shown increased numbers of partially vaccinated children residing in urban areas.^15^^,^^37^^–^^41^

Our findings indicate a high number of partially vaccinated children in Kampala.

We found that children from less poor households were more likely to be fully vaccinated than children from poor households. Despite efforts to prioritize vaccination programs in hard-to-reach areas characterized by households of low economic status and poor and vulnerable populations, coverage is still low. These findings are similar to other studies that underscore poverty level or socioeconomic position as a critical determinant of complete vaccination, with the odds of vaccination being higher among the less poor.^37^^,^^40^ Our findings also showed that more than half of the children were partially vaccinated, regardless of whether they resided in slum or nonslum areas. In contrast, previous studies showed that unimmunized children were mostly found among the urban poor living in slum areas, an outcome attributed to challenges accessing basic health services.^39^^,^^42^^,^^43^ Recent government and partner efforts have focused on providing vaccination services in informal settlements; however, in line with Immunization Agenda 2030,^7^ a need exists to also target partially immunized residents in nonslum areas to increase coverage and equity.

We found that coverage was highest for vaccines administered at birth (BCG and polio0), 6 weeks (DPT1, PCV1, and polio1), and 10 weeks (DPT2, PCV2, and polio2). High vaccine coverage for vaccines administered before 10 weeks indicates good access to vaccination services. However, coverage in the current study dropped for subsequent vaccine doses delivered at 14 weeks, including DPT3, PCV3, and polio3, indicating poor utilization of vaccination services in Kampala. Coverage for vaccines routinely administered simultaneously, such as DPT1 and Polio 1, had discrepancies, suggesting missed opportunities for vaccination. The prioritization of DPT performance by health workers may partly explain the differences in coverage as it is a key indicator tracked by UNEPI and other immunization stakeholders, as highlighted by evidence in Nepal and Mozambique.^44^^,^^45^ In other countries, including Uganda, overreporting of the DPT performance indicator has been attributed to Gavi immunization services support funding being aimed at increasing coverage of essential vaccines such as DPT.^46^ Differences in coverage for subsequent doses of vaccines are consistent with previous surveys conducted in Uganda and studies conducted in other countries such as Kenya and India.^17^^,^^40^^,^^41^^,^^47^^,^^48^ In similar settings, the discrepancies in coverage for doses that should be delivered at the same time have been partly attributed to health workers’ expressed lack of confidence to administer 2 vaccines at once owing to fear of contraindication and to their reluctance to open vials until enough children are present for vaccination to avoid wasting doses.^40^^,^^49^

High vaccine coverage for initial vaccines indicates good access to immunization services, but decreased coverage for subsequent vaccines suggests poor utilization of the services.

Our findings show that the timeliness of vaccination in Kampala has declined compared with 2010 estimates. A survey conducted in Kampala in 2010 reported that only 45.6% of all children in Kampala received complete vaccination within the recommended time range.^15^ Evidence in similar settings also shows significant delays in administering vaccines within their recommended time ranges, especially in developing countries.^48^^,^^50^^,^^51^ Delayed vaccine delivery creates a pool of children with incomplete or no immunity, which creates the risk of outbreaks of vaccine-preventable diseases. The timeliness of vaccines is important to prevent adverse effects and mortality, especially if certain vaccines are administered simultaneously or in reversed order.^52^^–^^54^ This emphasizes the need for vaccinations to be given on time according to the vaccination schedule.

### Drivers of and Barriers to Vaccination in Kampala City

**FIGURE 3:**
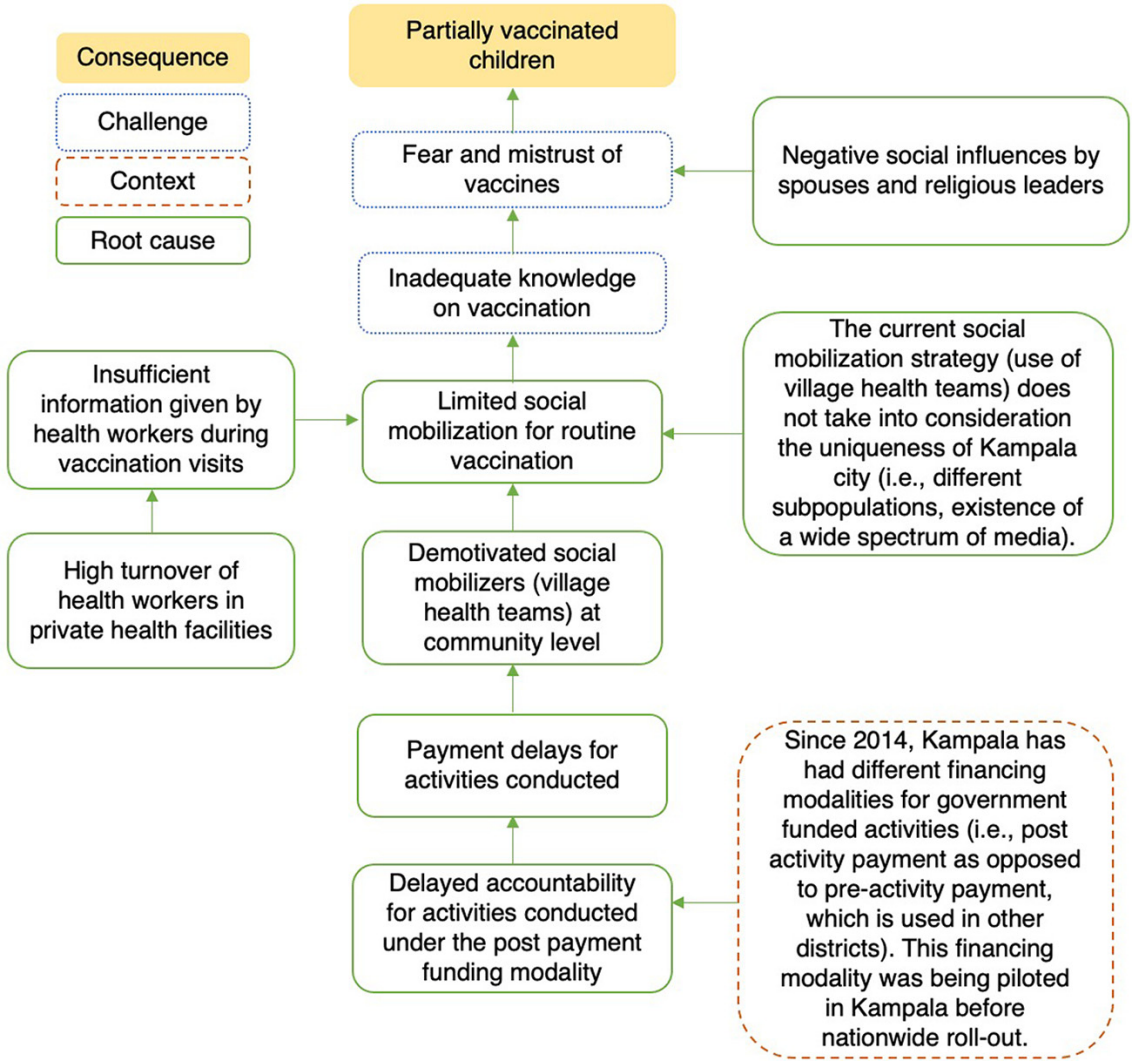
Root Cause Analysis for Caregivers’ Lack of Knowledge on Vaccination, Kampala, Uganda

Information on vaccination and its benefits was a key driver for completing the vaccination schedule among fully vaccinated children. However, several parents of partially vaccinated children stated that they were given inadequate information. Therefore, we constructed a root cause analysis on caregivers’ lack of knowledge on vaccination, which highlighted 2 factors in particular (Figure 3). First, parents reported that health workers gave insufficient information about vaccination benefits and the vaccination schedule during health facility visits. According to the immunization program at the Ministry of Health, high staff turnover rates of trained/mentored health workers exacerbate inadequate information transfer, especially in private health facilities. Secondly, UNEPI lacks a fit-for-purpose communication strategy that considers the complexities of the urban context. Instead, it uses a uniform communication strategy in rural and urban areas that capitalizes on the traditional social mobilization structures for routine vaccination, with little consideration of the urban context. The limited social mobilization for routine vaccination can also explain the inadequate knowledge. Respondents at national and district levels reported limited social mobilization for routine vaccination despite being included in the annual work plan. Social mobilization efforts were more pronounced during nonroutine vaccination campaigns conducted during new vaccine introductions and child health days. A possible explanation is that social mobilizers may be demotivated owing to an inadequate budget for social mobilization activities and delays in payment. Additionally, the current mobilization strategy overlooks the uniqueness of Kampala, including the existence of different subpopulations and the availability of a broad spectrum of media.

Information on immunization was a key driver for completing the immunization schedule, and not everyone received the information.

Other studies have also shown that health workers often provide insufficient information on vaccination to parents.^41^^,^^55^^,^^56^ Further, a crucial determinant of vaccination is knowledge about the benefits of vaccination and the vaccine schedule, especially in urban areas.^41^^,^^49^^,^^57^^–^^60^ Improving patient-health worker interaction and communication during vaccination visits positively affects the vaccination experience of parents as well as health-seeking behaviors.^61^^,^^62^ In the absence of adequate information on vaccination, parents are influenced by the perceptions of spouses, family, and community members, which may be inaccurate. These findings suggest the need for continuous health education efforts about the benefits of vaccination and the importance of completing the vaccine schedule. However, these efforts should be tailored to the complexities of urban settings characterized by mobile and diverse populations, busy parents, and a lack of formal addresses. Previous research shows that education programs designed in collaboration with the target community can effectively increase coverage uptake, especially in slum populations.^63^

Another barrier to vaccination in this study was vaccine stock-outs that may have been caused by rationing vaccines due to the limited stock at the national level. According to providers’ perceptions, the limited stock was a result of inadequate funds to procure sufficient vaccines. Additionally, we found that vaccine stock-outs may also be explained by the challenges and delays in the distribution of vaccines in Kampala from satellite to lower-level health facilities. Vaccine stock-outs have also been reported in Kampala, with shortages of polio vaccines attributed to poor management.^21^ Similarly, vaccine stock-outs are common in other urban settings, as shown by the frequent shortage of the polio vaccine.^49^ The underlying causes for vaccine stock-outs in Kampala are not clear, but given that vaccine shortages lead to incomplete and untimely vaccinations, stock-outs need to be further investigated.

Other barriers to vaccination were vaccine stock-outs and long waiting times at health facilities.

Our study findings also revealed that long waiting times prevented parents from vaccinating their children. According to key informants and FGDs, the long waiting times occur because of delayed commencement of vaccination sessions and health workers’ reluctance to open multiple-dose vaccine vials until enough children are present to avoid wastage. Long waiting times in Kampala have previously been attributed to the late arrival of health workers, especially at public health facilities.^21^ Long waiting times affect the quality of vaccination services due to poor patient satisfaction, which impairs access to health services, especially in light of busy schedules and employment obligations in the urban context.^20^^,^^61^ Similarly, delays in opening vaccine vials to decrease wastage have also been documented in other countries, with health workers only opening vials when the number of children is equal to or greater than half of the number of doses per vial.^49^^,^^64^ One study showed that vaccinators wait for at least 6 children before opening a measles vaccine in Nigeria, leading to parents having to leave and thus opportunities for vaccination being missed.^65^ The Uganda immunization program encourages health workers to open multidose container vaccines for every eligible child, but this guidance is not always followed. According to respondents at the community level, if the requisite number of children fell short, parents were asked to return later, which deterred them from immunizing their children.

Participants also reported hidden costs, such as payment for vaccination cards and other requirements, despite the official national policy that vaccination services are free of charge. Similarly, informal costs associated with vaccination, especially in private health facilities, have been reported in Kampala, with study participants reporting that they had to pay between US$0.2 and US$4 for vaccination services.^21^ Financial deprivation is a significant hindrance to vaccination. Parents often lack money for necessities such as food, let alone transportation to health facilities.^20^ Informal payments for health services are common, especially in developing countries. This practice negatively affects health care quality, discouraging patients from seeking health services.^66^^,^^67^ Although parents are often willing to pay for services they perceive to be high quality, not all parents can afford to pay.^68^ Hidden costs deter and exclude the urban poor from receiving vaccination services, but most of their children are at least partially immunized. These findings call for the engagement of health workers in public and private facilities to address this issue. A primary health care model that harnesses the power of the private sector is needed.

Participants also reported hidden costs, despite the official national policy that immunization services are free of charge.

A key strength of this study was its mixed-methods approach using quantitative and qualitative methods. We collected household vaccination data in all the divisions of Kampala, considering income poverty levels. We also conducted FGDs and IDIs at the community level to determine why parents partially vaccinate their children in an urban setting. Finally, we triangulated study findings across data collection methods (quantitatively and qualitatively) and by level (UNEPI at the Ministry of Health, at the district and community levels).

### Limitations

Despite these strengths, our study had some limitations. First, we defined our sampling frame based on income, poverty levels, and the number of measles outbreaks in 2017. In addition, the vaccination coverage did not include individuals without vaccination cards. As such, our findings might not represent the entire population in Kampala as the coverage may be underestimated or overestimated. However, our estimates are close to UDHS estimates. Second, while this study focused on the demand-side factors affecting vaccination coverage in Kampala, several supply-side issues emerged from our data, including vaccine stock-outs, long waiting times, and hidden costs. We also did not adequately explore the intricacies of the delivery of vaccination services by the private sector. This illustrates the difficulty in disentangling the demand side from the supply side since the way care is delivered affects uptake and demand. Subsequent research will address these issues in greater detail. Finally, this being a household survey among residents of Kampala means that our results do not capture the mobile population that comes to Kampala daily, yet they are the majority. Innovative survey methods that also capture the highly mobile nonresidents in urban settings need to be developed.

## CONCLUSIONS

We found that many children are partially vaccinated, with most vaccinations not received on time. The main driver to completing the vaccination schedule was parents’ appreciation of the benefits of vaccination. Parents with partially vaccinated children faced barriers to vaccination arising from inadequate information about vaccination, vaccine stock-outs, long waiting times at health facilities, and hidden vaccination costs. Increasing vaccine knowledge requires a deliberate communication strategy for routine vaccination in Kampala coupled with targeted social mobilization efforts tailored to the complexities of urban settings. Additionally, the public-private partnership in urban areas needs to be strengthened to address the challenges of long waiting times and hidden costs and increase access.

Furthermore, there is a need to target children in slum areas and nonslum areas as they could also potentially be unvaccinated or under-vaccinated. These findings inform UNEPI and its stakeholders; other EPI groups, especially in developing countries; and the Gavi Alliance partners. These findings were used to inform the development of the urban health strategy that includes vaccination. Given the huge daytime mobile population that comes to major urban settings, innovative survey methods that capture vaccination coverage in both resident and nonresident populations need to be developed. Future research should investigate the supply-side drivers of vaccination, including the frequent vaccine stock-outs highlighted in this study.

## Notes

**Peer Reviewed**

**First published online:** August 12, 2022.
